# Reference long-read isoform-aware transcriptomes of 4 human peripheral blood lymphocyte subsets

**DOI:** 10.1093/g3journal/jkac253

**Published:** 2022-09-26

**Authors:** Cassandra R Woolley, Julia H Chariker, Eric C Rouchka, Easton E Ford, Elizabeth A Hudson, Sabine J Waigel, Melissa L Smith, Thomas C Mitchell

**Affiliations:** Department of Microbiology and Immunology, University of Louisville School of Medicine, Louisville, KY 40202, USA; KY INBRE Bioinformatics Core, University of Louisville, Louisville, KY 40202, USA; Department of Neuroscience Training, University of Louisville, Louisville, KY 40202, USA; KY INBRE Bioinformatics Core, University of Louisville, Louisville, KY 40202, USA; Department of Biochemistry and Molecular Genetics, University of Louisville School of Medicine, Louisville, KY 40202, USA; Department of Microbiology and Immunology, University of Louisville School of Medicine, Louisville, KY 40202, USA; Department of Biochemistry and Molecular Genetics, University of Louisville School of Medicine, Louisville, KY 40202, USA; Department of Biochemistry and Molecular Genetics, University of Louisville School of Medicine, Louisville, KY 40202, USA; Department of Biochemistry and Molecular Genetics, University of Louisville School of Medicine, Louisville, KY 40202, USA; Department of Microbiology and Immunology, University of Louisville School of Medicine, Louisville, KY 40202, USA; Brown Cancer Center, University of Louisville Health, Louisville, KY 40202, USA

**Keywords:** isoform, lymphocyte, B cell, T cell, NK cell, RNA-Seq, Iso-Seq, transcriptome, illumina, pacific biosciences

## Abstract

Long-read sequencing technologies such as isoform sequencing can generate highly accurate sequences of full-length mRNA transcript isoforms. Such long-read transcriptomics may be especially useful in investigations of lymphocyte functional plasticity as it relates to human health and disease. However, no long-read isoform-aware reference transcriptomes of human circulating lymphocytes are readily available despite being valuable as benchmarks in a variety of transcriptomic studies. To begin to fill this gap, we purified 4 lymphocyte populations (CD4^+^ T, CD8^+^ T, NK, and Pan B cells) from the peripheral blood of a healthy male donor and obtained high-quality RNA (RIN > 8) for isoform sequencing and parallel RNA-Seq analyses. Many novel polyadenylated transcript isoforms, supported by both isoform sequencing and RNA-Seq data, were identified within each sample. The datasets met several metrics of high quality and have been deposited to the Gene Expression Omnibus database (GSE202327, GSE202328, GSE202329) as both raw and processed files to serve as long-read reference transcriptomes for future studies of human circulating lymphocytes.

## Introduction

Structural details of transcript isoforms, also called transcript variants, are important to catalog to advance understanding of the roles they play in cellular fitness. With the isoform sequencing (Iso-Seq) method, individual mRNA molecules are first converted into cDNA and then into circular templates, which undergo multiple rounds of iterative sequencing using Single Molecule Real-Time (SMRT) technology (Pacific Biosciences). Ultimately, this generates a highly accurate (>99.9%) intramolecular consensus read of each full-length mRNA transcript (Sharon *et al.* 2013; Wenger *et al.* 2019). Iso-Seq has the potential to identify important changes in isoform structure, such as those occurring in activated and/or memory human lymphocytes participating in the immune response. However, to robustly characterize functional changes in RNA biology in the context of innate and adaptive immunity, a reference transcriptome is needed to serve as a benchmark. Thus far, most human Iso-Seq transcriptomes currently published have included stem cells and cancer lines or are pooled broadly from multiple tissues (Au *et al.* 2013; Sharon *et al.* 2013; Xie *et al.* 2018; Kuo *et al.* 2020). To our knowledge, no Iso-Seq transcriptome has been published for primary human B, T, or NK cells, despite the fact that isoform-specific expression patterns are known to play a role in defining lymphocyte development and functions (Yabas *et al.* 2015; Peng *et al.* 2022). To begin to fill this gap, we purified these 4 lymphocyte subsets from the peripheral blood of a healthy donor to obtain high-quality RNA (RIN > 8) for SMRT sequencing and Iso-Seq analysis. Full-length nonconcatemer (FLNC) reads as well as processed Iso-Seq data files have been deposited to the Gene Expression Omnibus (GEO) database (Edgar *et al.* 2002) alongside matched data from Illumina short-read RNA-Seq that was performed in parallel (GSE202329 SuperSeries). Many novel transcript isoforms supported by both Iso-Seq and RNA-Seq data were identified. Each cell-type sample and its corresponding sequence data set met several metrics of quality as assessed by flow cytometric and SQANTI3 (Tardaguila *et al.* 2018) analysis, respectively, indicating that they will be valuable as benchmarks for future studies.

## Methods and materials

### Human blood collection and PBMC isolation

Venous blood, 175 ml, from a healthy consented 57-year-old male donor was collected in and adjusted to 6 mM K_3_EDTA using standard phlebotomy. Collection was approved by the University of Louisville Institutional Review Board under expedited review (IRB 14.0661).

PBMCs were isolated using Sepmate PBMC Isolation Tubes (StemCell Technologies, cat no. 85450) as previously described (Woolley *et al.* 2022), with an additional granulocyte depletion step (RosetteSep Human Granulocyte Depletion Cocktail, StemCell Technologies, cat no. 15624) as directed by the manufacturer. Cell yield was determined using the count per microliter feature of a Cytek Northern Lights flow cytometer as previously described (Woolley *et al.* 2022), immediately after which the PBMCs were further processed to purify blood lymphocyte subsets.

### Human lymphocyte subset enrichment

Lymphocyte subsets were purified from freshly prepared PBMC using EasySep (StemCell Technologies) negative magnetic selection kits: Human CD4^+^ T Cell Isolation Kit (StemCell Technologies, cat no. 17952), Human CD8^+^ T Cell Enrichment Kit (StemCell Technologies, cat no. 19053), Human NK Cell Enrichment Kit (StemCell Technologies, cat no. 19055) and Human Pan-B Cell Enrichment Kit (StemCell Technologies, cat no. 19554), per manufacturer’s instructions. After purification, 2 million (Pan B) or 3 million (CD4^+^ T, CD8^+^ T, and NK) isolated cells were lysed in Buffer RLT Plus (RNeasy Plus Mini Kit, Qiagen, cat no. 74134), per the manufacturer’s protocol, and lysates were kept at 4°C until RNA extraction. The remaining isolated cells were used for flow cytometric staining of markers to assess cell viability and purity as described below.

### Flow cytometric staining and analysis

Isolated cells were distributed at 0.2 million cells per well in a 96-well plate for flow cytometric staining (Woolley *et al.* 2022) with some modifications. For viability staining, cells were first washed twice with PBS lacking calcium and magnesium (PBS^−/−^) and then resuspended in 100 µl of PBS^−/−^ containing eBioscience Fixable Viability Dye eFluor 780 (ThermoFisher, cat no. 65-0865-14). Cells were incubated for 30 min prior to wash and resuspension in PBS^−/−^ followed by fixation with 1% formaldehyde and transfer to 12 mm × 75 mm flow cytometry tubes. Fixed cells were kept on ice until flow cytometric analysis.

To evaluate purity of the lymphocyte subset samples, cells were washed twice with standard stain buffer (PBS^−/−^ with 0.09% NaN_3_ and 2% human serum) prior to resuspension in a cocktail of antibodies specific for markers of myeloid and lymphoid lineages. The antibody cocktail was generated by first adding appropriate amounts of fluorescent-conjugated monoclonal antibodies (Table 1) to Brilliant Stain Buffer Plus (BD Biosciences, cat no. 566385), and subsequently adding standard stain buffer to reach a cumulative 100 µl per test. After resuspension in this antibody cocktail, cells were incubated for 30 min prior to wash and resuspension in stain buffer. Cells were then fixed with 1% formaldehyde and transferred to flow cytometry tubes which were kept on ice until flow cytometric analysis.

**Table 1.** Antibody markers used for cell-type identification.

Supplier, catalog #	Marker specificity	Cell type defined								
		***CD4^+^ T***	CD4^+^ NK T	***CD8^+^ T***	CD8^+^ NK T	CD4−CD8− T	***NK***	***Pan B***	CD14^+^ CD16^−^ monocyte	CD14^+^ CD16^+^ monocyte
BD, 560367	CD45	**+**	**+**	**+**	**+**	**+**	**+**	**+**	**+**	**+**
ThermoFisher, 58-0038-42	CD3	**+**	**+**	**+**	**+**	**+**	**−**	**−**		
BioLegend, 300534	CD4	**+**				**−**		**−**		
ThermoFisher, 64-0088-42	CD8			**+**	**+**	**−**		**−**		
BD, 564057	CD56		**+**		**+**		**+**	**−**		
BD, 563522	CD43							**+** ^a^		
ThermoFisher, 69-0199-42	CD19							**+** ^a^		
Tonbo, 20-0149-T100	CD14							**−**	**+**	**+**
BD, 555408	CD16							**−**	**−**	**+**

A fraction of each purified sample was stained with antibodies specific for the myeloid and lymphocyte lineage markers listed. Bolded and italicized cell types are the lymphocyte subsets purified. Cell types were defined within the samples by flow cytometric analysis as follows: +, the presence of the lineage marker was required; −, cells with the lineage marker were excluded from the group; no symbol entry means the marker was not considered in defining the cell type.

a Pan B cells were defined as positive for CD19 or CD43 or both, and negative for myeloid, T, and NK lineage markers.

**Fig. 1.**
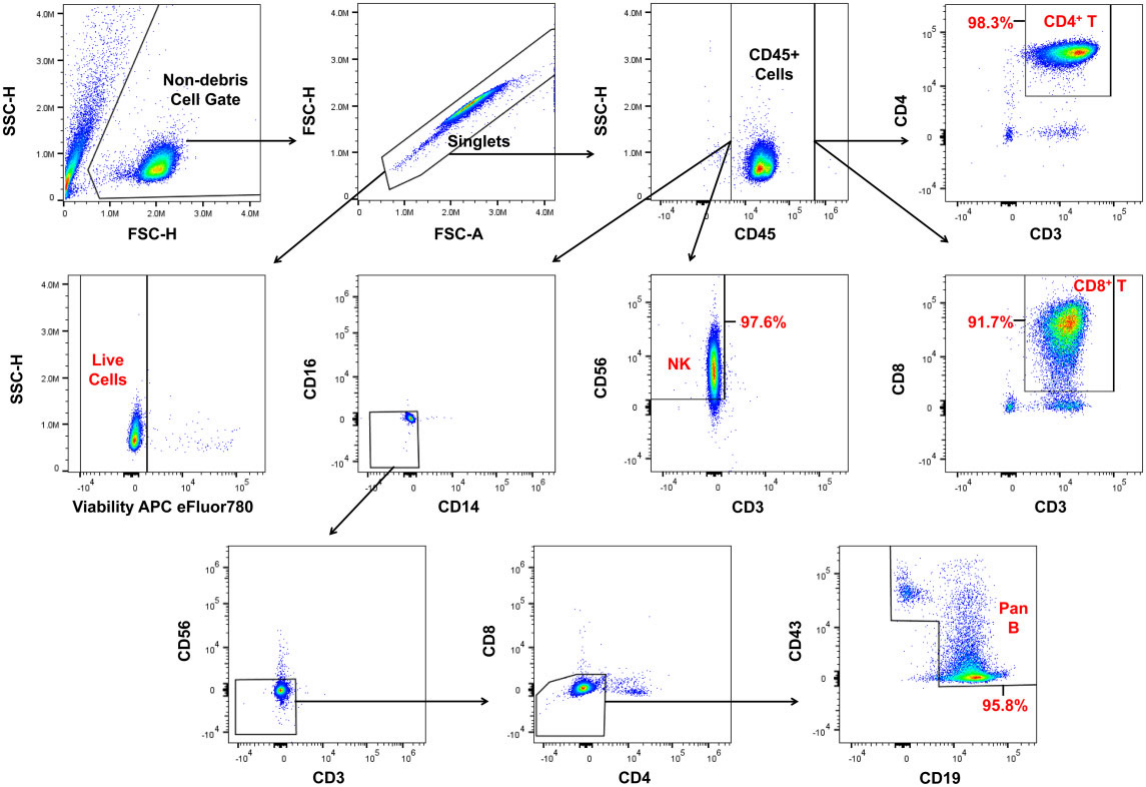
Gating strategy used to define live cells and major subsets within each purified cell sample. A fraction of each purified sample was stained with makers specific for myeloid and lymphoid lineages and the gating strategy depicted was used to define live cells, CD4^+^ T cells, CD8^+^ T cells, NK cells, and Pan B cells. Inset percentages show the final purity of each respective lymphocyte population. Dot plots are representative of gating used upstream of these populations. In calculating viability, the fraction of live cells of total singlets was used. In calculating purity of the desired cell type, the fraction of the corresponding cell type of total CD45^+^ cells was used.

Flow cytometry was performed with a Cytek Northern Lights 3-laser flow cytometer, and spectral profiles of each fluorophore were unmixed using SpectroFlo software (Cytek Biosciences) and appropriate single-stain and unstained controls to account for autofluorescence. Processed data files were analyzed in FlowJo (BD Biosciences). Purity and viability were determined using the gating strategies shown in Fig. 1. Purity is reported as the average of 2 technical replicates, and viability as a single replicate value, of a fraction of the same cells used for RNA isolation.

### RNA extraction and purification

Total cellular RNA was extracted and purified from 2 to 3 × 10^6^ cells per sample using RNeasy Plus Mini Kit (Qiagen, cat no. 74134) following the manufacturer’s protocol. RNA from each lymphocyte subset sample was eluted in 30 µl of RNA-ase free water and kept in a cold block on ice until Iso-Seq library preparation the same day. Remaining RNA was frozen and kept at −80°C for RNA-Seq library preparation.

### Iso-Seq library preparation, sequencing, and initial data analyses

To generate Iso-Seq libraries, 500 ng of high-quality RNA (RIN > 8) was used as initial input into oligo-dT primed cDNA synthesis using commercially available NEB Next reagents (New England Biolabs, cat no. E6421L). Barcoded primers were incorporated into the cDNA during second-strand synthesis. Following double-stranded cDNA amplification, transcripts were equimolar pooled to include 2 samples per SMRTbell library preparation (CD4^+^ and CD8^+^ T; NK and Pan B). SMRTbell libraries were generated from the pooled cDNA as recommended by the manufacturer. Briefly, the pooled cDNA underwent enzymatic DNA damage and end repair prior to ligation with SMRTbell hairpin adaptors. Final libraries were purified with magnetic beads prior to annealing to sequencing primer (v4) and binding to polymerase (v2.1). Sequencing was performed using 1 SMRTcell 8M per pair of barcoded samples on a Sequel IIe system in the UofL Sequencing Technology Center. Following data generation, multiple, iterative sequences covering a single SMRTbell molecule were collapsed to generate highly accurate circular consensus sequence (CCS) reads, followed by analysis using the IsoSeq 3 tool in the SMRTLink software suite (v10.1). CCS reads were further filtered on those CCS reads with quality >99%, producing “HiFi” reads. The IsoSeq 3 pipeline demultiplexed HiFi reads per individual samples from the pooled sequencing data, filters out amplification artifacts, trims primers and polyadenylation (polyA) tails, and produces de novo FLNC transcripts for downstream mapping and annotation.

### Tertiary Iso-Seq data analysis and isoform characterization

A custom pipeline was developed to integrate steps in the cDNA Cupcake protocol for postprocessing of Iso-Seq v3 clustered FLNC reads (https://github.com/Magdoll/cDNA_Cupcake). The alignment files for the FLNC reads were converted to fastq (Cock *et al.* 2010) and fasta format. Fasta files were aligned to the *Homo sapiens* reference genome assembly (hg38) using minimap2 v2.18-r1015 (Li 2018). The resulting alignment files were collapsed into isoforms based on the sequence similarity using the cDNA Cupcake Python script collapse_isoforms_by_sam.py. Isoform abundance was calculated using get_abundance_post_collapse.py, and isoforms were filtered to include those with at least 2 supporting reads with filter_by_count.py*.* Isoforms were also filtered to remove possible nonfull-length reads by removing those with indications of a degraded 5′ prime region, i.e. apparent 5′ shortened isoforms with otherwise equivalent long reads (filter_away_subset.py). SQANTI3 (Tardaguila *et al.* 2018) was used to summarize the results for each of the individual samples.

### RNA-Seq library preparation and sequencing

RNA-Seq libraries were prepared using the Universal Plus mRNA-Seq with NuQuant (NuGEN, cat no. MO1485). For each sample, 100 ng of RNA (in a volume of 50ul) was used for poly A enrichment. First- and second-strand cDNA was synthesized followed by adapter and unique index ligation. Samples were barcoded using the Universal Plus (UDI) 96-Plex Adaptor Plate (NuGEN, cat no. S02480). The concentration of each library was measured using a Qubit dsDNA HS kit (Thermo Fisher, cat no. Q32854). The correct size of each library was confirmed by Agilent Bioanalyzer analysis using the DNA High Sensitivity Kit (Agilent Technologies, cat no. 5067-4626). In addition, correct adapter and index ligation, as well as the library concentration, was validated by sequencing all libraries on a MiSeq Nano Kit V2 300 cycles (Illumina, cat. no. MS-103-1001). 1.5 pM of barcoded library was denatured, and sequencing was performed on an Illumina NextSeq 500 using the NextSeq 500 75 cycles High Output Kit v2.5 (Illumina, cat no. 20024906). One single-end replicate was performed for each sample. The MiSeq quality control run, as well as the data run on the Illumina NextSeq 500 yielded reads with a data quality of 94.9% of reads at or above Q30. The read alignment rate to the human reference genome Hg38 was 98.6% or higher for each sample.

### RNA-Seq data analysis

The quality of the sequenced reads was assessed using FastQC (v.0.10.1, Andrews 2015) which indicated high-quality reads such that no sequence trimming was necessary. The sequences were directly aligned to the *H. sapiens* reference genome assembly (hg38) using the STAR aligner (v2.6, Dobin *et al.* 2013) 2-pass method with Gencode (v39) annotations (Frankish *et al.* 2019). When used to filter Iso-Seq long reads, RNA-seq short reads were restricted to uniquely mapped genomic positions by setting the STAR aligner option outFilterMultimapNmax to 1.

### Integration of Iso-Seq and RNA-Seq

To assess short-read coverage across the splice junctions identified in the long-read data, short reads were realigned using the STAR 2-pass method with the reference GTF produced by the cDNA Cupcake analysis. The resulting SJ.out.tab file was provided as input to SQANTI3 (v4.0), which produced a file with short-read coverage across each splice junction in the long reads (junctions.txt). Additional SQANTI3 input included the cDNA Cupcake GTF, Gencode (v39) annotations, the hg38 assembly, long-read transcript abundance, a polyA motif list and a cap analysis gene expression (CAGE) peak (TSS sites) BED file for human and mouse. A custom script was written to identify isoforms with at least 1 short read covering all internal splice junctions (not including the first and last junctions) using the junction file produced by SQANTI3. Coverage of only internal junctions was used for filtering since we saw a pattern of noticeably less short-read coverage of long-read transcript ends, particularly 5′ ends, similar to as previously observed by others (Tardaguila *et al.* 2018).

Transcripts not meeting the filtering criteria were removed from the GTF, resulting in a filtered GTF with high confidence transcripts based on short-read coverage. The filtered GTF was then annotated with SQANTI3. To make visual comparisons across the 2 methods, UCSC Genome Browser tracks were created to explore expression across the genome (Kent *et al.* 2002). The custom tracks for Iso-Seq and RNA-Seq data were created using guidelines and utilities available on the UCSC Genome Browser website. Custom tracks are available to view at http://genome.ucsc.edu/cgi-bin/hgHubConnect?hgHub_do_redirect=on&hgHubConnect.remakeTrackHub=on&hgHub_do_firstDb=on&position=chr1:206,903,317-206,921,941&hubUrl=http://162.215.210.70/~tracks/Mitchell_Lymphocyte_Reference_Transcriptomes/hub.txt.

### Functional annotation for novel isoforms

Ensembl gene IDs were extracted for all isoforms classified as novel in catalog (NIC) and novel not in catalog (NNC) from the SQANTI3 classification file for each cell type. The IDs were used in functional annotation analysis, performed separately for individual cell types, with gProfiler2 (Kolberg *et al.* 2020). WikiPathways was selected as the primary annotation source. Disease-related pathways were excluded on account of samples being from a healthy donor, and only pathways that returned an adjusted *P*-value of less than 0.01 in at least one of the cell-type samples were reported. Expression levels were not considered for this analysis.

## Results and discussion

### Quality of purified cell samples

Purified cells used to extract RNA for sequencing were sampled and found to be highly viable (viability ≥91%, Table 2). CD4^+^ T, NK, and Pan B cells showed excellent purity (purity >95%, Table 2), suggesting that RNA extracted from these samples was also reasonably exclusive of other cell types. CD8^+^ T cells, on the other hand, were less pure (91.7%), with the major contaminating cell types being CD4^−^CD8^−^CD3^+^ cells (6.8%, Table 2) and Pan B cells (1.4%, Table 2). Thus, sequencing data obtained for the CD8^+^ T cell sample, though still highly reflective of the cell type, should be considered less specific.

**Table 2.** Viability and purity of samples.

Purified sample name	% live cells	% cell type						
		CD4^+^ T (CD4^+^NK T)	CD8^+^ T (CD8^+^NK T)	CD4^−^ CD8^−^ CD3^+^	NK	Pan B	CD14^+^ CD16^−^ monocyte	CD14^+^ CD16^+^ monocyte
CD4+ T	**99.1**	**98.3** (0.4)	^a^	0.9	^a^	0.6	^a^	^a^
CD8^+^ T	**99.2**	^a^	**91.7** (3.9)	6.8	0.3	1.4	0.1	^a^
NK	**98.3**	^a^	^a^	^a^	**97.6**	0.8	^a^	^a^
Pan B	**91.0**	^a^	^a^	0.3	1.5	**95.8**	0.1	^a^

Four lymphocyte populations were purified by negative selection and a fraction was set aside for flow cytometric analysis. % live cells, percent of events in a live gate among nondebris singlets. % cell type, percent of each cell type among all CD45^+^ cells. See Table 1 for cell surface markers used. Bold text entries indicate viability and purity of the cell types targeted for purification and sequencing.

a Less than 0.1%.

The lymphocytes used in this study were purified by negative selection to avoid transcriptional artifacts caused by antibody binding. Because our goal was to generate reference transcriptomes that were broadly representative of subsets within each circulating lymphocyte population, purification kits with fewer exclusions were chosen whenever possible. For example, the Pan B-cell kit was selected because it did not exclude CD43^+^ cells, a marker of plasma B cells (Table 3, CD19^+^ and/or CD43^+^ B-cell frequencies), and the CD4^+^ and CD8^+^ T-cell purification kits did not exclude CD4^+^CD56^+^ or CD8^+^CD56^+^ NK T cells, respectively (Table 2, NK T-cell frequencies reported among CD45^+^ cells alongside purity). Furthermore, based on flow cytometric data from 2 previous experiments, the peripheral blood donor possessed a range of circulating naive and memory T-cell subtypes which we viewed as desirable for these reference data sets (Table 4). Ultimately, all 4 isolated populations were high-quality and representative samples of their respective lymphocyte subset.

**Table 3.** Frequencies of B-cell subtypes in each sample.

Purified sample	% of CD45^+^ cells			% of Pan B cells		
	CD19^+^ CD43^−^	CD19^+^ CD43^+^	CD19^−^ CD43^+^	CD19^+^ CD43^−^	CD19^+^ CD43^+^	CD19^−^ CD43^+^
CD4^+^ T	0.3	0.1	0.2	61.6	9.1	29.3
CD8+ T	^a^	0.2	1.2	0.51	11.7	87.8
NK	^a^	^a^	0.8	0.76	2.3	96.9
Pan B	88.1	4.4	3.1	92.2	4.6	3.3

Calculated by flow cytometric analysis of a fraction of cells in each of the purified samples that were sequenced, reported both out of all CD45^+^ cells within a sample as well as out of the Pan B subset in the sample.

a Below cutoff value of 0.1%.

**Table 4.** Frequencies of T-cell subsets within the peripheral blood of the donor selected.

T-cell type	% subset			
	Naïve (CD62L^+^ CD45R0^−^)	Tcm (CD62L^+^ CD45R0^+^)	Tem (CD62L^−^ CD45R0^+^)	CD62L^−^ CD45R0^−^
CD4^+^	**56.1 **±** **3.6	**34.2 **±** **3.7	**9.1 **±** **0.1	**0.6 **±** **0.3
CD8^+^	**36.8 **±** **0.8	**21.3 **±** **2	**34.1 **±** **0.5	**7.8 **±** **1.7

Reported as the average percent ± standard error of the mean based on flow cytometric analysis in 2 prior experiments, 3 technical replicates per experiment, using the same male donor. Subset percentages are reported out of the total CD4^+^ or CD8^+^ T-cell parent population within peripheral blood mononuclear cells. Tcm, T central memory; Tem, T-effector memory. % subset, percent among CD4^+^ or CD8^+^ T-cell parent population.

### RNA-Seq reads and genomic mapping

RNA-sequencing generated ∼102–124 million short reads per lymphocyte population, and when allowing the reads to map to multiple loci the alignment rates were above 98% for all samples (Table 5). To parallel the parameters used for Iso-Seq analyses, we also mapped RNA-seq reads to hg38 in a manner that restricted their alignments to a single locus. Alignment rates for these uniquely mapped reads were between 85% and 89% (Table 5). The high alignment rates achieved with both mapping strategies indicated the RNA-Seq datasets were of high quality.

**Table 5.** Total RNA-Seq reads and alignment.

Sample	Total reads	Multimapped		Uniquely mapped	
		Aligned reads	Alignment rate (%)	Aligned reads	Alignment rate (%)
CD4^+^ T cells	102,630,137	101,328,169	98.73	86,939,812	84.71
CD8^+^ T cells	124,630,896	123,279,274	98.91	108,717,331	87.23
NK cells	114,296,338	113,001,142	98.86	101,944,728	89.19
Pan B cells	112,101,017	110,586,693	98.64	95,317,079	85.02

Multimapped parameters allow reads to map to 1 or multiple places across the genome. Uniquely mapped parameters allow only mapping to a single locus.

### IsoSeq sequencing and initial data metrics

To generate Iso-Seq transcriptomes, pairs of barcoded samples were loaded on each of 2 8M SMRTcells and sequenced on the Sequel IIe system, which produced ∼1–2 million FLNC reads per lymphocyte population. SMRTcell loading, read length, and yield metrics fell within expected ranges for both SMRTcells (Table 6). For data derived from both SMRTcells, 50% of bases were in reads >110,000 (read length N50), allowing >30 passes of the average 3-kb mRNA, which resulted in highly accurate HiFi data (Q38, 99.98%) as the input into IsoSeq analysis. These metrics were consistent with the generation of highly accurate and extensive sequencing data for all 4 lymphocyte populations.

**Table 6.** Number of FLNCs, clusters, and nonzero transcripts for Iso-Seq.

Sample	SMRTcell pool	Quality loading efficiency (P1) (%)	Read length N50 (bp)	Number of HiFi reads	Mean HiFi read quality	Number of FLNC
CD4^+^ T cells	1	65.7	120,750	2,805,160	Q38 (99.98%)	1,495,624
CD8^+^ T cells						976,691
NK cells	2	93.1	113,750	4,325,837	Q38 (99.98%)	2,179,981
Pan B cells						2,091,657

### Iso-Seq transcript annotation

After processing HiFi reads into FLNCs, the mRNA transcripts they represented were categorized and summarized using SQANTI3, provided any given transcript had been observed as at least 2 unique molecules. The 4 lymphocyte populations expressed 11,542–14,487 genes with 34,211–59,845 distinct isoforms, as determined by the locations of splice junctions and transcription start and stop boundaries (Table 7). The number of annotated genes ranged from 10,402 (CD8^+^ T cells) to 12,162 (B cells) across the individual samples. The 961–2,325 novel genes (Table 7) were categorized as such because they had no annotated counterparts in GENCODE v39 and may represent Ig or TCR gene rearrangements, although several hundred monoexon transcripts mapped to novel locations in the genome. The 15,433–33,918 novel transcripts identified (Table 7) were categorized as such because they did not match any known Gencode v39-annotated transcript.

**Table 7.** Number of genes and isoforms detected by Iso-Seq.

Sample	Unique genes	Unique isoforms	Annotated genes	Novel genes	Novel transcripts
CD4^+^ T cells	11,973	44,159	10,962	1,011	22,248
CD8^+^ T cells	11,542	34,211	10,402	1,140	15,433
NK cells	12,176	50,905	11,215	961	27,119
Pan B cells	14,487	59,845	12,162	2,325	33,918

### Iso-Seq results after filtering for short-read coverage

To reduce the risk of artifacts, the genes and isoforms detected by Iso-Seq alone (Table 7) were filtered to include only those with at least 1 RNA-Seq short read for every internal splice junction present in a transcript. As shown in Table 8, the exclusion of long-read transcript sequences lacking short-read support reduced the number of unique isoforms detected to 60% of the value observed before filtering. This reduction occurred more frequently with annotated genes than novel genes because 92% of the latter remained after filtering (an average of 1,255 novel genes detected after, as compared to 1,359 before, filtering). However, only 55% of novel transcripts remained after filtering (an average of 13,563 novel transcripts detected after, as compared to 24,680 before, filtering), suggesting filtering effectively refined the dataset, as desired, while retaining most novel isoforms.

**Table 8.** Number of genes and isoforms detected by Iso-Seq after RNA-Seq filtering.

Sample	Unique genes	Unique isoforms	Annotated genes	Novel genes	Novel transcripts
CD4^+^ T cells	7,797	23,524	6,904	893	10,647
CD8^+^ T cells	8,417	22,304	7,326	1,091	9,380
NK cells	9,052	32,555	8,178	874	15,730
Pan B cells	10,649	34,858	8,488	2,161	18,496

### Classification of transcript isoform structures

**Fig. 2.**
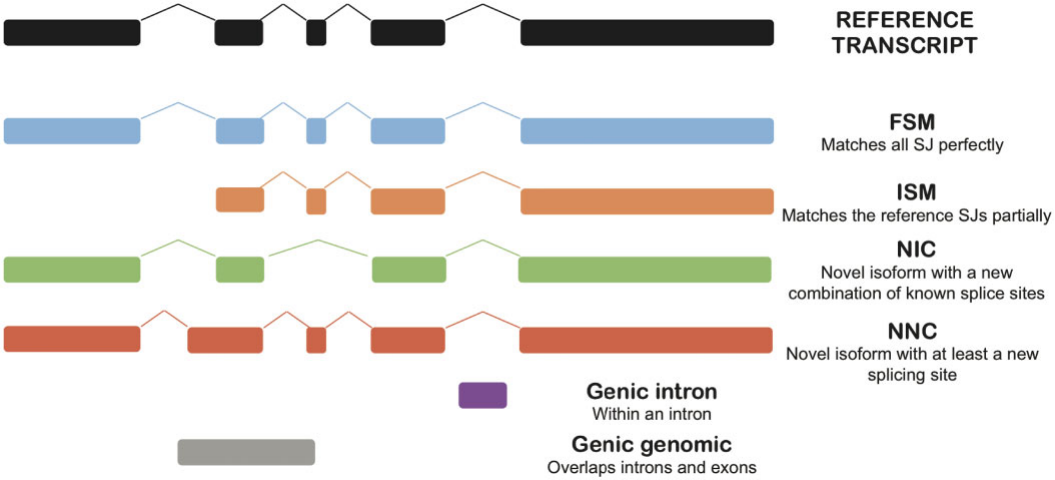
Schematic of SQANTI3 isoform structural characterization (image taken from https://github.com/ConesaLab/SQANTI3/wiki/SQANTI3-isoform-classification:-categories-and-subcategories, Tardaguila *et al*., 2018). FSM, full splice match; ISM, incomplete splice match; NIC, novel in catalog; NNC, novel, not in catalog. Intron retention events are not depicted but would fall within ISM, NIC, NNC, or fusion categories.

Filtered transcript isoforms were classified by SQANTI3 into several structural categories, based on alignment to previously reported reference transcripts, and the usage of known donor and acceptor splice sites. These were either full splice match (FSM), incomplete splice match (ISM), NIC, novel NNC, intergenic (between annotated genes), antisense (anti-sense to an annotated gene), fusion (fusion of 2 annotated genes), genic genomic, or genic intron (Fig. 2). Most isoform structures matched their reference transcripts completely (FSM), but many novel transcript structures were also identified within each sample (e.g. NIC and NNC, Table 9).

**Table 9.** Number of transcripts identified in each structural category after filtering by short-read coverage.

Sample	FSM	ISM	NIC	NNC	Genic genomic	Intergenic	Antisense	Fusion	Genic intron	Intron retention^a^
CD4^+^ T cells	10,998	1,879	4,524	4,406	603	544	393	177	0	2103
CD8^+^ T cells	11,066	1,858	4,296	3,290	494	720	399	181	0	1757
NK cells	14,254	2,571	7,281	6,642	663	553	365	225	0	3075
Pan B cells	13,359	3,003	6,492	6,283	998	1,510	742	2,470	1	3189

a Intron retention subcategory events were tabulated from within ISM, NIC, NNC, and fusion categories.

**Fig. 3.**
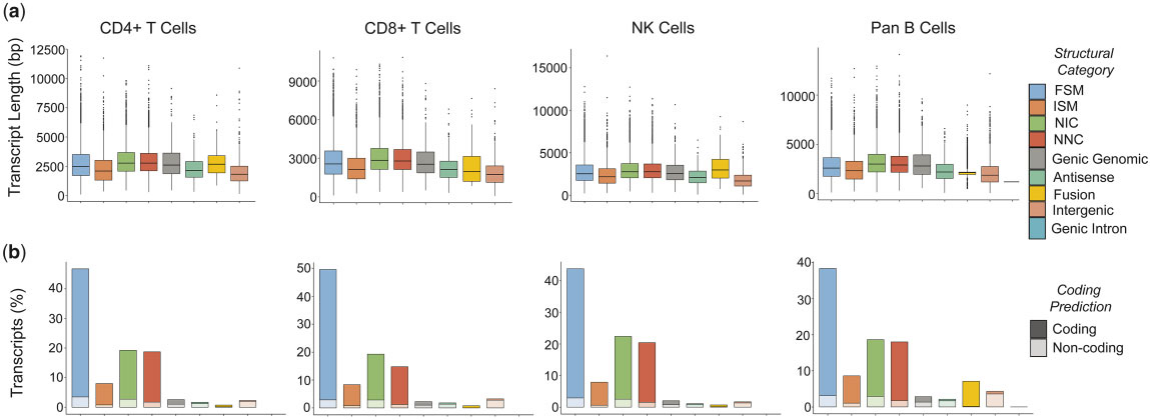
Characteristics of transcripts within each structural category. From left to right, each graph represents a different lymphocyte subset. a) Box-and-whisker plots depict the distribution of transcript lengths (bp) for each transcript structural category. Center lines of the plots represent median transcript lengths. b) Bar graphs depict the proportions of transcripts predicted to be protein-coding. Bar height indicates the % of transcripts within each structural category out of the total transcripts present in the sample. Darker or lighter shading within a bar represents the proportion of the category predicted to be protein-coding or noncoding, respectively.

Across all samples, the median values and distribution of lengths in novel (NIC and NNC) isoforms were similar to those of corresponding transcripts with complete or partial reference matches (FSM and ISM) (Fig. 3a). Furthermore, most novel transcripts identified were predicted to correspond to protein-coding transcripts (Fig. 3b). Though these transcripts remain to be fully validated, these attributes support the notion that many novel transcripts identified in our sequence datasets are likely real variants of known transcripts with potential functional relevance in protein expression.

**Fig. 4.**
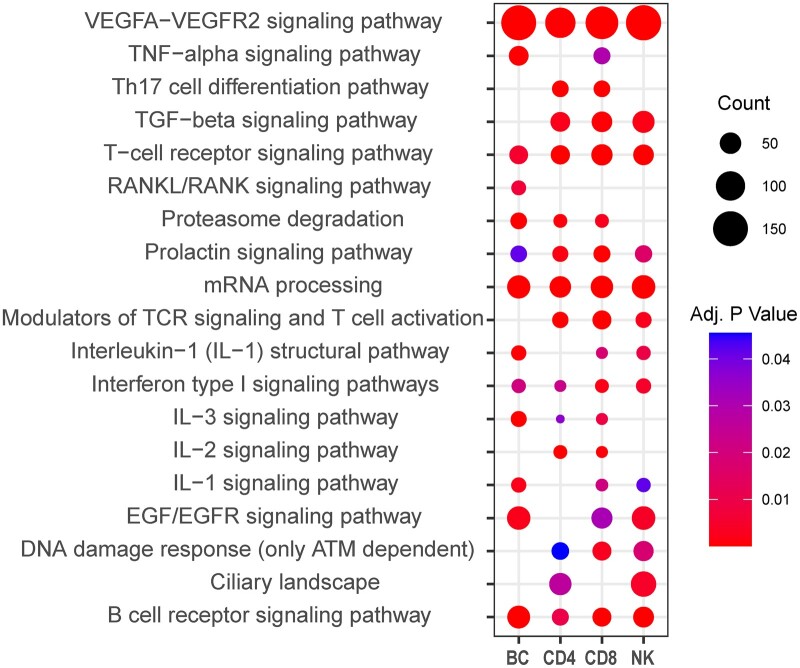
WikiPathways enriched for novel isoforms. Dot plot depicting gene-level functional classification and enrichment for novel isoforms in each lymphocyte subset. The size of the dot represents gene count within a category while heatmap-based coloration indicates *P*-value adjusted for false positive expectation. Only nondisease pathways that returned an adjusted *P*-value of <0.01 in at least one of the cell-type samples are depicted.

Novel transcripts (NIC and NNC) were primarily enriched within immune-important signaling pathways (Fig. 4), further supporting the potential for relevance to the field. However, enrichment here does not directly mean a pathway was active, as many cell-type-specific pathways share common genes, such as T- and B-cell receptor signaling sharing *GRB2*. It is also important to consider that isoform functionalities can be vastly different, even for the same gene (Brett *et al.* 2002), so the roles of novel isoforms may not align with their assigned gene-level functional classifications. Still, pathways enriched for novel isoforms, and particularly those differentially enriched between samples such as IL-2 signaling, could have yet unexplored layers of cell-specific isoform-level regulation. Further functional validation of novel isoforms is necessary before more accurate annotation and enrichment analyses can be performed.

### Quality metrics of filtered isoforms

**Fig. 5.**
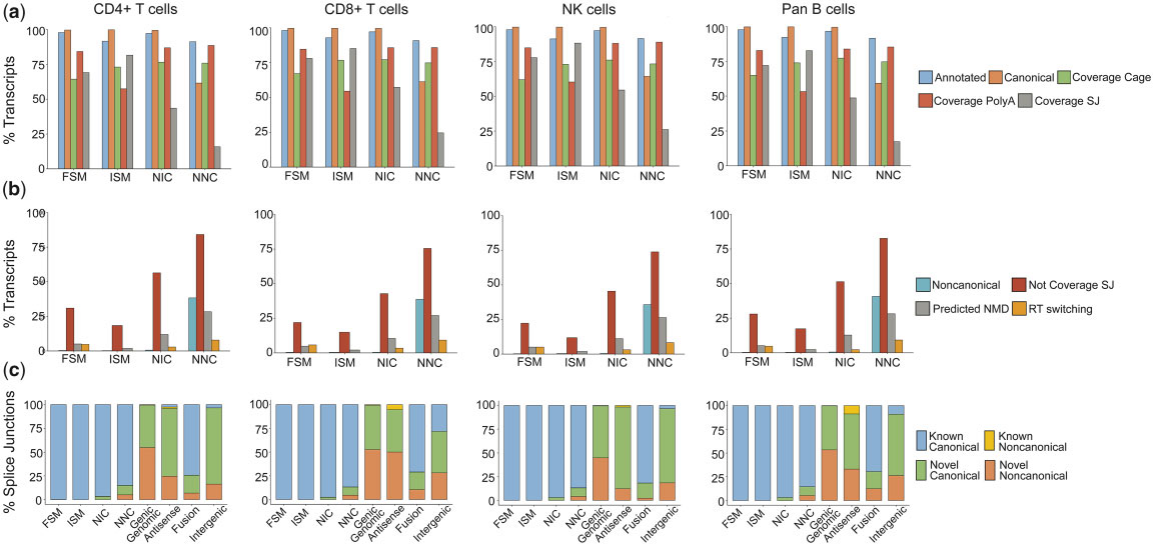
Quality control metrics within each isoform sample dataset. From left to right, each graph represents a different lymphocyte subset. a) Metrics of good quality, the % of transcripts within the noted categories which have: Annotated, mapped to annotated genes; Canonical, all canonical splice junctions; Coverage Cage, an identified CAGE Peak; Coverage PolyA, an identified polyA motif; Coverage SJ, supporting short-read coverage of all splice junctions. b) Metrics of bad quality, the % of transcripts within the noted categories which have: Noncanonical, at least 1 noncanonical splice junction; Not Coverage SJ, no short-read coverage of at least 1 splice junction; Predicted NMD, predicted nonsense-mediated decay; RT switching, predicted RT switching occurrence. c) Proportions of all splice junctions identified which are: known canonical, known noncanonical, novel canonical, or novel noncanonical.

Using the top 4 isoform categories (FSM, ISM, NIC, and NNC) to assess metrics of quality for each sample dataset via SQANTI3, we observed relatively high percentages of transcripts within each category demonstrating good quality attributes and low percentages of transcripts with bad quality attributes (Fig. 5, a and b, respectively). For example, over 90% of transcripts in all categories mapped to known annotated genes, and the majority had CAGE peak and polyA motif support (Fig. 5a). On the other hand, low fractions of transcripts were predicted to have undergone nonsense-mediated decay (NMD) or reverse transcriptase (RT) switching, both common causes of artifactual novelty (Fig. 5b).

Compared to the unfiltered transcriptomes, filtering for short-read coverage of internal splice junctions increased the fraction of transcripts for which all splice junctions were supported by short-read data (Fig. 5a, unfiltered data not shown). Yet, for all categories within our filtered dataset, there were a fraction of transcripts with no short-read coverage for at least 1 splice junction. This is likely due to our filtering based on only internal splice junctions rather than all splice junctions, as was considered for this metric. This fraction was particularly high for the novel, not-in-catalog (NNC) transcripts where 73.8–84.2% of transcripts contained at least 1 splice junction without short-read coverage (Fig. 5b). A high number of our NNC transcripts also contained at least 1 noncanonical splice junction (35.7–40.8%, Fig. 5b). However, when considering the overall number of noncanonical splice junctions out of all junctions in these transcripts, the percentage is much lower (all samples <10% noncanonical junctions with the rest being novel canonical or known canonical, Fig. 5c), suggesting that this characteristic may be reflective of the NNC classification itself rather than the transcript quality.

Taken together with the high quality of each contributing cell sample, these metrics support the notion that the filtered datasets provided here consist of representative and high-quality polyadenylated transcripts, some entirely novel and previously unannotated. These will add to the known catalog of transcript isoforms present in healthy circulating B, T, and NK cells and will be valuable for reference in future lymphocyte transcriptomic analyses.

## Data Availability

All Sequencing data sets were deposited to the GEO. For each Iso-Seq human lymphocyte dataset, raw FLNC files were deposited in BAM format alongside processed data files (Iso-Seq only: GSE202328, SuperSeries: GSE202329). The processed files consist of raw gene isoform counts, annotation (GTF) files, and UCSC Genome Browser tracks. The annotation files were obtained prior to and after filtering for RNA-Seq coverage. Raw Illumina RNA-Seq data for each dataset were deposited as fastq.gz files (RNA-Seq only: GSE202327, SuperSeries: GSE202329). Processed RNA-Seq files deposited consist of raw gene counts and UCSC Genome Browser tracks. Custom UCSC Genome Browser tracks created using both Iso-Seq and RNA-Seq data are available through http://genome.ucsc.edu/cgi-bin/hgHubConnect?hgHub_do_redirect=on&hgHubConnect.remakeTrackHub=on&hgHub_do_firstDb=on&position=chr1:206,903,317-206,921,941&hubUrl=http://162.215.210.70/~tracks/Mitchell_Lymphocyte_Reference_Transcriptomes/hub.txt. The authors affirm all data necessary for confirming the conclusions of the article are present within the deposited sets, article, figures, and tables.
